# Isolated dextrogastria with eventration of right hemidiaphragm and hiatal hernia in an adult male

**DOI:** 10.1186/s12876-022-02127-x

**Published:** 2022-02-10

**Authors:** Shahid Aziz, Simone König, Haseeb Noor, Muhammad Nadeem, Rabaab Zahra, Faisal Rasheed

**Affiliations:** 1 BreathMAT Lab, Pakistan Institute of Nuclear Science and Technology, Islamabad, Pakistan; 2 Department of Microbiology, Faculty of Biological Sciences, Quaid-I-Azam University, Islamabad, Pakistan; 3 IZKF Core Unit Proteomics, University of Münster, Münster, Germany; 4 Department of Gastroenterology, Federal Government Polyclinic Hospital, Islamabad, Pakistan; 5 Department of General Surgery, Services Institute of Medical Sciences, Lahore, Pakistan

**Keywords:** Isolated dextrogastria, T2-IDG, Hiatal hernia, Gastritis, Visceral transposition, Barium test, CT scan

## Abstract

**Background:**

Type 2 isolated dextrogastria in conjunction with protrusion of the right hemidiaphragm and hiatal hernia is an uncommon anomaly among all transpositions of the viscera. Clear diagnosis is not straightforward in such cases both clinically and with various imaging techniques leaving often only laparotomy for diagnosis.

**Case presentation:**

Here, we discuss the case of a so far asymptomatic 19-year-old male, who had a 3-month history of abdominal pain and 2 days of vomiting with absolute constipation, and reduced air entry in the base of the right lung. A large air fluid level was found in the right lower hemithorax, furthermore, a loss of the normal diaphragmatic outline, and paucity of the bowel gases in the rest of the abdomen. Computer tomography with contrast was suggestive of loss of right lung volume, with stomach and bowel loops herniating into the right hemithorax and compressive atelactatic changes in the adjacent lung alongside an enlarged liver. A barium test showed the stomach fundus and body posteriorly positioned, while both duodenal bulb loops and the duodeno-jejunal junction alongside the small and large bowels were detected in their normal positions.

**Conclusion:**

In case of visceral transpositions, routine diagnostic blood and radiological tests may lead the health care provider to misdiagnosis. It is necessary, in particular when surgery is required, to carefully elucidate the organ anomaly. The use of additional imaging and radiological methods may be called for; CT scan and a barium test were critical here. This is the first case of isolated dextrogastria with eventration of right hemidiaphragm and hiatal hernia reported from Pakistan providing insights for diagnostic procedures.

## Background

Isolated situs inverses is an unusual incidence and typically involves the heart, a condition known as innate dextrocardia. Total viscerum is infrequent having a prevalence of 1/6000 to 1/8000 cases [1]. Among all the visceral transpositions, isolated dextrogastria (IDG) together with natural position of both thoracic and abdominal viscera is rare [1–5] and has been reported in two different forms, IDG-type 1 (T1) and T2, with T1, having a prevalence < 1 in 100,000, being less frequent than T2 [6]. In T1-IDG, the stomach lies behind the liver, while in T2, the stomach is above and protrusion of the right diaphragm is associated (for a great introduction to IDG see ref. [1]). All other viscera are in their regular position resulting in malfunction of the foregut to normally rotate (T1-IDG) or failure of its descent from the chest (T2-IDG) [1, 3, 7]. Routine chest radiography in patients of T1-IDG identifies the normal left-side gastric gas shadow on the right side, while patients of T2-IDG show lung pathology in clinical and radiological presentation [7, 8]. T2-IDG with protrusion can mimic other anomalies including right-sided hiatal hernia and lobar pneumonia leading to diagnostic dilemmas [1]. IDG patients may remain asymptomatic and are only incidentally diagnosed during radiological workups, abdominal surgery or autopsies [1, 2, 8–10], but serious cases of antenatally diagnosed IDG have also been reported [3]. The latter study is a retrospective analysis from Southampton Hospital, UK, discussing 20 newborn cases from a 10-year span (2004–2014); it also serves as excellent introductory reading into the topic. Five more IDG cases of children from different locations can be found in the literature [7, 11–13] as well as a report on 447 infants and children with anomalies of rotation and fixation [14]. IDG with protrusion of the right hemidiaphragm and subsequent volvulus has hardly been published [4, 14, 15]. Here we present such a case in a 19-year-old male from Pakistan.

## Case presentation

**Fig. 1:**
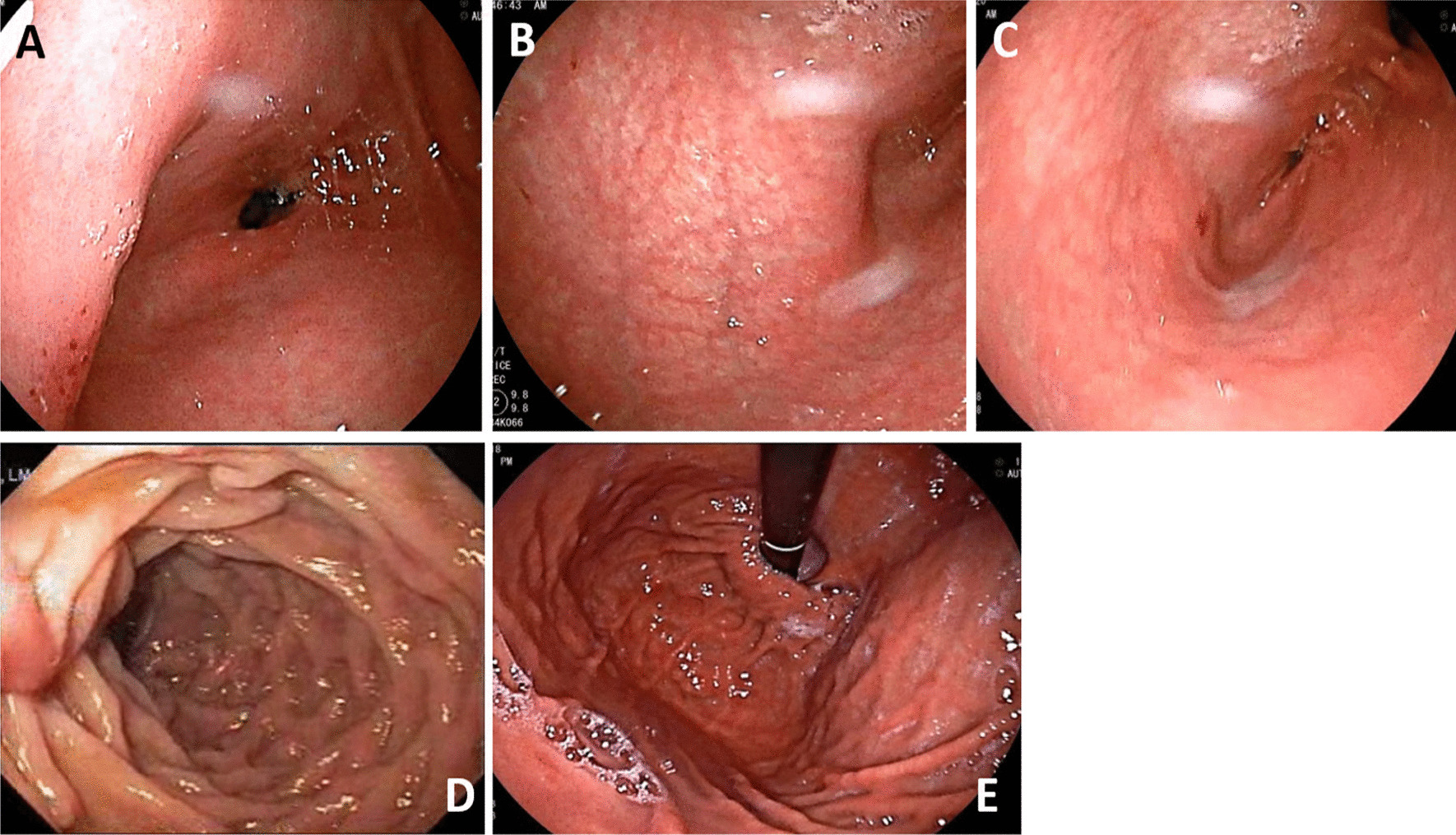
Upper gastroduodenal endoscopy showed antral gastritis (**A**), corpus gastritis (**B**), pan gastritis (**C**), duodenitis (**D**) and hiatal hernia (**E**)

The patient presented to his health care practitioner at tertiary care hospital with a history of 3 months of abdominal pain, 2 days of vomiting with absolute constipation, and reduced air entry in the base of the right lung. He had no history of *Mycobacterium tuberculosis*, Hepatitis B or C infection, addiction, or allergy to any medicine, and he had been asymptomatic up to this point. Based on the clinical symptoms and suspicion of liver abscess, he was admitted and treated with antibiotics and intravenous (IV) fluids. His symptoms did not improve so that radiological investigations (X-ray, ultrasound and computer tomography (CT) scans of chest, abdomen, and pelvis with contrast) were performed. Furthermore, the patient was subjected to blood tests (complete blood picture, renal and liver function tests, serum electrolytes, and coagulation profile). The patient was also advised for endoscopic evaluation. The endoscopist faced difficulties during endoscopic advancement. On arriving at cardia, the fundus was found in abnormal position on the right side and a greater curvature was running in an abnormal way. The endoscope had to be maneuvered (rotation and advancement) into the direction opposite to that of normal anatomy. The endoscopist detected the altered anatomy as a result of the congenital problems and gastric volvulus, as well as inadequate inflation of stomach. Moreover, endoscopy revealed erythema in esophagus, stomach, and duodenum along with hiatal hernia (Fig. 1). Biopsies were taken from stomach and duodenum for histopathology, which showed chronic antral, corpus, and pan gastritis along with duodenitis without evidence of intestinal metaplasia, dysplasia, and *Helicobacter pylori*. The patient was subsequently treated with proton pump inhibitors and antacids for 7 days.

**Fig. 2:**
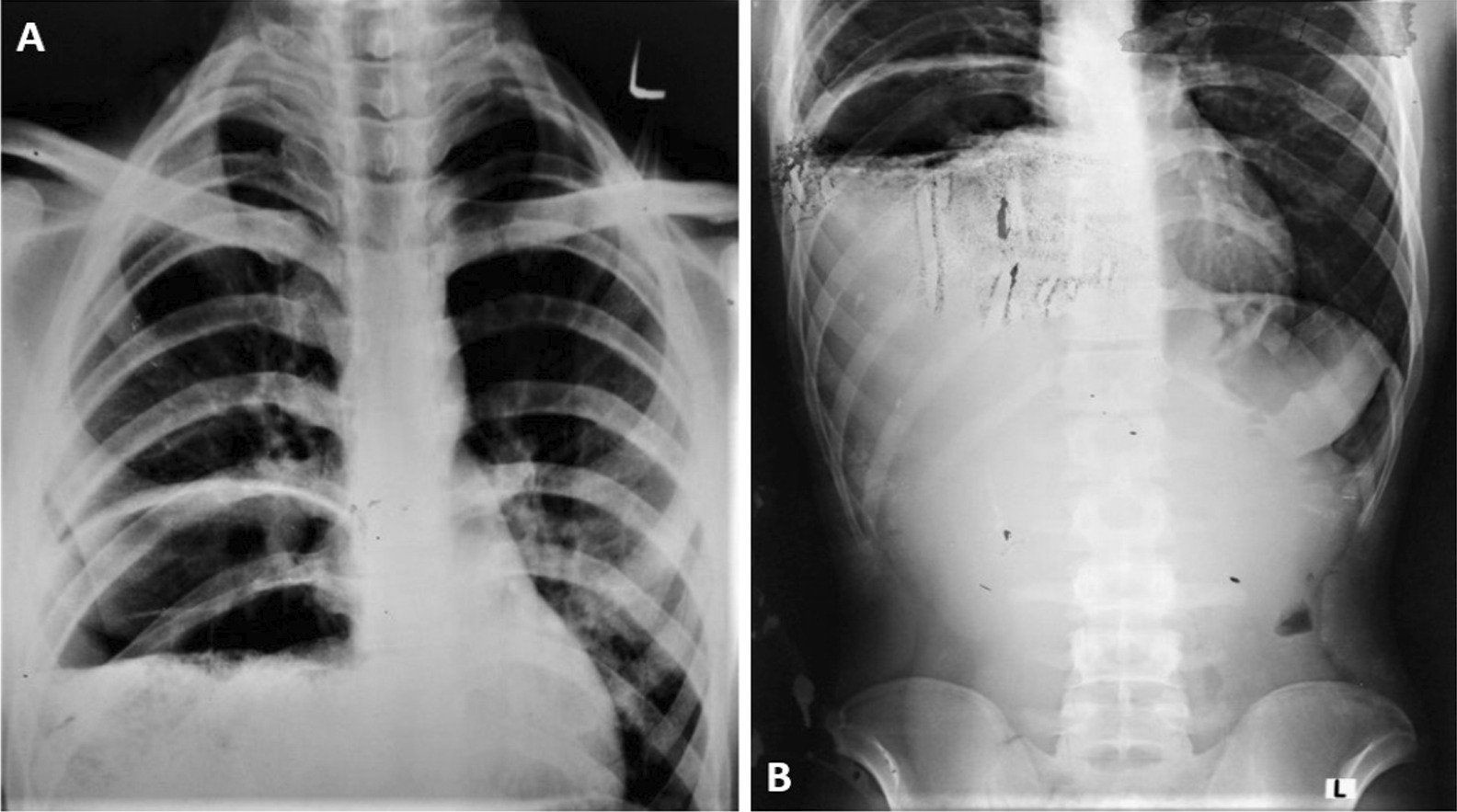
Chest X-ray revealed no gastric air shadow (**A**), but a large air fluid level in the right lower hemithorax with loss of normal diaphragmatic outline and paucity of bowel gases in the rest of the abdomen (**B**)

During the chest X-ray, no gastric air shadow was seen as expected from a normal left side position of the stomach (Fig. 2A). Additionally, a large air-fluid level was observed in the right lower hemithorax along with the loss of the normal diaphragmatic outline, which was likely hollow viscous. Paucity of the bowl gases was noted in the rest of the abdomen (Fig. 2B).

**Fig. 3:**
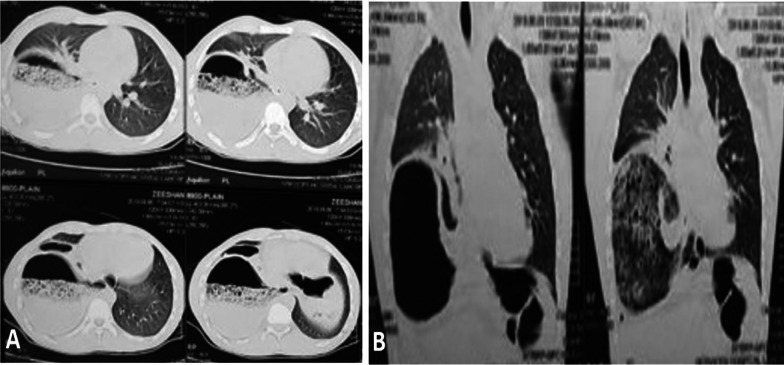
Contrast enhanced CT images of chest and abdomen visualized the loss of right lung volume along with stomach and bowel loops herniating into the right hemithorax. **A** Axial view of CT scan of chest and abdomen showed atelectatic changes in lung, normal heart size and hollow viscus on right with air and fluid alongside the enlarged liver. **B** Coronal view presented large cavitating lesion below right lung with loss of right lung volume and opacifying with contrast

**Fig. 4:**
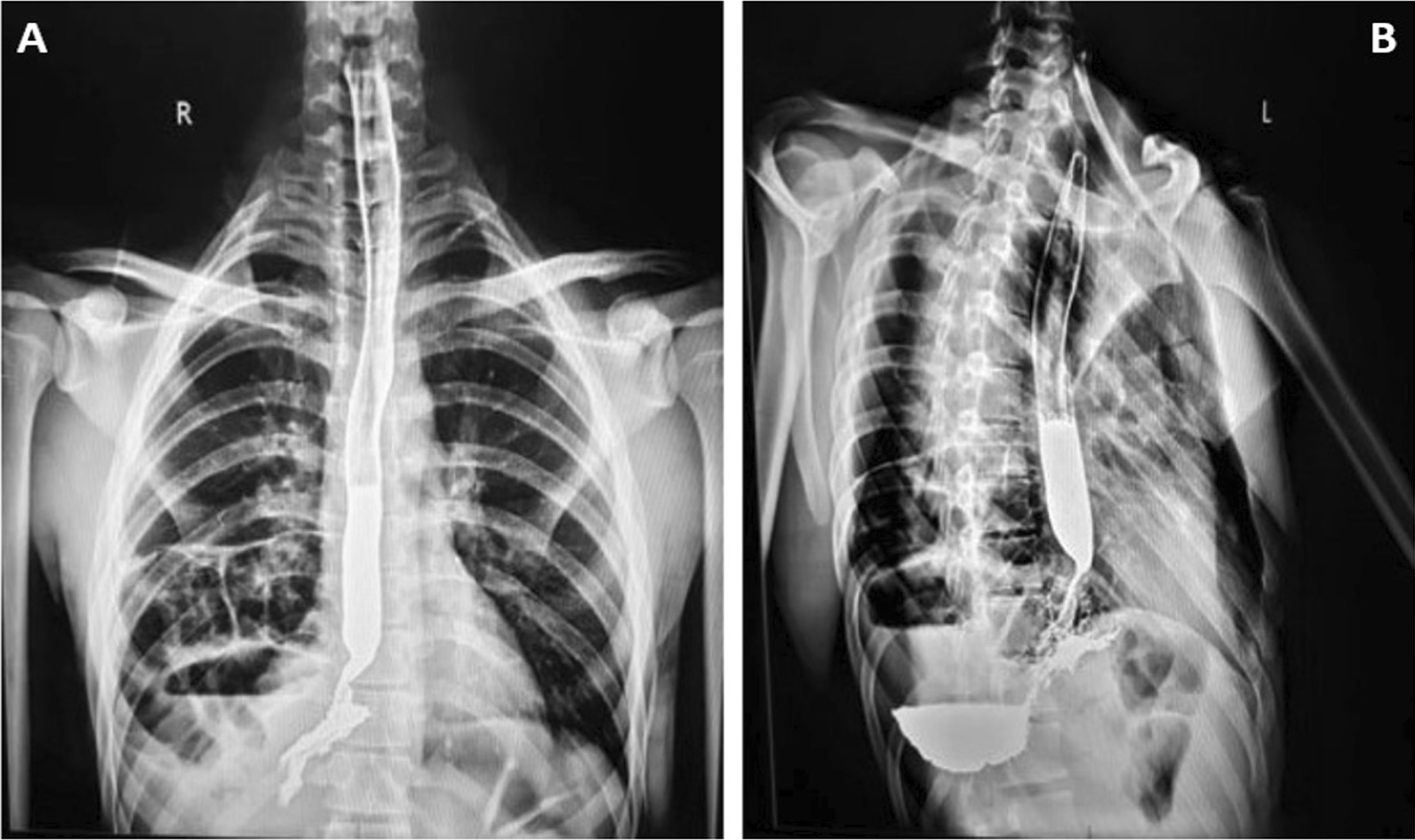
Barium test visualized the actual position of the stomach lying above the liver. **A** The front contrast radiograph showed the stomach lying on the right side of chest with normal opacification of esophagus and a cavitating lesion on the right side below the lung. **B** The lateral view of contrast radiograph confirmed the finding visualizing the stomach filled with contrast agent at the distilled end

Contrast CT scan of chest and abdomen visualized the loss of right lung volume along with stomach and bowel loops herniating into the right hemithorax (Fig. 3). Moreover, compressive atelectatic changes were observed in the adjacent lung alongside the enlarged liver (Fig. 3). In order to ascertain the cause of vomiting, a barium test was performed, which located the stomach above the gastro-esophageal junction and in the right hemithorax. It was normally opacified with contrast (Fig. 4). The stomach fundus and body were posteriorly positioned, which appeared as a mirror image on the frontal projection, while both duodenal bulb loops and the duodeno-jejunal junction alongside the small and large bowel were found in their normal positions. Barium was well tolerated; the patient did not report any vomiting or other symptoms with its ingestion. All the blood tests were normal except the white blood cell count (Table 1).

**Table 1:** Results of blood tests

Blood screen	Hb	WBC	Platelets
	12.6 g/dl	18,180/mm^3^	170,000/mm^3^
Renal parameter tests	Urea	Creatinine	
	32 mg/dl	0.9 mg/dl	
Liver function tests	ALT	AST	
	17 U/l	21 U/l	
Serum electrolytes	Na	K	
	132 mmol/l	3.6 mmol/l	
Coagulation profile	PT	APTT	INR
	14 s	33 s	1.1 ratio

Hb, hemoglobin; WBC, white blood cell count; ALT, alanine transaminase; AST, aspartate transaminase; Na, sodium; K, potassium; PT, prothrombin time; APTT, activated partial thromboplastin time; INR, international normalized ratio

A nasogastric tube was passed to examine the suspected intestinal obstruction. Both before and after the investigation the patient was given IV antibiotics (injections ceftriaxone 500 mg (*bd*), metronidazole 500 mg (*tds*), omeprazole 40 mg IV (*od*) and IV fluids). He improved symptomatically and was subsequently monitored for delayed complications. The patient was eventually discharged after 7 days of final diagnosis of isolated dextrogastria with the advice for regular follow up every month.

## Discussions and conclusions

This case report describes T2-IDG in a 19-year-old boy, who presented to his health care practitioner with gastrointestinal complications including 3 months of abdominal pain, 2 days of vomiting with absolute constipation, and reduced air entry in the base of the right lung. IDG is a rare occurrence and its diagnosis thus not straightforward. In both types of IDG, T1 and T2, patients remain asymptomatic and the anomaly is usually discovered incidentally [1]. For instance, authors reported the absence of normal left sided gastric gas shadow in IDG-T1 in radiological findings [11]. In comparison with IDG-T2, they found more abnormalities including right sided hiatal hernia along with other pathological complications, which may also mimic abscess. In other work, the barium test showed gastric fundus and corpus situated behind and appeared as mirror image on the frontal projection [2, 8, 9]. Moreover, the duodenum bulb loop, which lies to the right of the abdominal wall, and the duodeno-jejunal junction, which acts as border between both duodenum and jejunum, along with large and small bowel, were seen in their normal positions.

In our case, the patient remained un-diagnosed throughout his childhood before the symptoms appeared as a young adult. Typically, IDG poses problems at an early age [3]; it remains unclear why our patient had no medical complaints during his childhood. As in other IDG cases [2, 7, 8, 11, 12], most blood tests were normal and the symptoms arose from organ misalignment. Only a series of radiological tests identified the cause of his symptoms, because IDG can mimic other possible complications including loculated hydropneumothorax, pleural effusion and subphrenic abscess [1].

The stomach has its fixed position at the oesophageal hiatus and pylorus junction secured by ligamentous attachment, which prevents its abnormal rotation. In the absence of anatomical anchors, the stomach moves under the protruded diaphragm predisposing it to gastric volvulus. This unusual association has only been seen in an infant [8], the eventration of the right hemidiaphragm and volvulus of a right-sided stomach in a newborn [6]. In a another research, authors found four cases of IDG reported in infancy but not in young children [2, 7]. Among them, only one sixth of the children having gastric volvulus had association with underlying protrusion of the hemidiaphragm [13].

In the present case, we report such rare association in a young man with T2-IDG and compressive atelectatic changes in the adjacent lung alongside the enlarged liver. T2-IDG belongs to a spectrum of conditions under flag of situs inverses and is usually associated with other anomalies. Prognosis mainly depends on the associated conditions. T2-IDG without any anomaly typically has an excellent prognosis.

The findings of this case report were corroborated with barium test and CT scan. These radiological findings for a T2-IDG case in Pakistan have not been described in literature to the best of our knowledge. The case will provide an excellent roadmap to the operating surgeons. In cases of gastrointestinal situs inversus, plain chest radiographs raise suspicion. CT scan and barium test may be then used to better define the situs inversus and its associated clinical complications. As barium is well tolerated, it also aids the consultants to manage patients with T2-IDG.

In cases of exceptional gastrointestinal abnormalities, routine blood tests and simple radiographs may direct the health care practitioner to false diagnoses [1, 9]. In fact, eventration of the right hemidiaphragm with asymptomatic IDG in a newborn could not be made clinically or by various imaging techniques and was ascertained at laparotomy [1]. In our patient, only CT and a barium study allowed the elucidation of the organ anomaly. The symptoms of the patient, gastritis, duodenitis and severe constipation—were not primarily caused, but certainly supported by the IDG and the hiatal hernia. The knowledge about his situs inverses will simplify future diagnoses in this patient considerably. Surgical intervention is not needed for T2-IDG when properly recognized. This is the first T2-IDG case report from Pakistan of in a 19-year-old male with a protrusion of right hemi-diaphragm along with a large air fluid level in the right lower hemithorax and the loss of the normal diaphragmatic outline with paucity of the bowel gases in the rest of the abdomen.

## Data Availability

Research data is available on request.

## Abbreviations

IDG: Isolated dextrogastria
T1/T2: Type 1/2
IV: Intravenous
CT: Computer tomography
Hb: Hemoglobin
WBC: White blood cell count
ALT: Alanine transaminase
AST: Aspartate transaminase
PT: Prothrombin time
APTT: Activated partial thromboplastin time
INR: International normalized ratio
